# 4126 Intermuscular adipose tissue secretes pro-inflammatory, extracellular matrix, and lipid signals related to insulin resistance and type 2 diabetes

**DOI:** 10.1017/cts.2020.73

**Published:** 2020-07-29

**Authors:** Darcy Kahn, Simona Zarini, Emily Macias, Amanda Garfield, Kathleen Harrison, Melanie Cree-Green, Jonathan Schoen, Bryan Bergman

**Affiliations:** 1 University of Colorado at Denver; 2 University of Colorado, Anschutz Medical Campus

## Abstract

OBJECTIVES/GOALS: Intermuscular adipose tissue (IMAT) has been associated with insulin resistance and type 2 diabetes, yet mechanistic studies addressing the functional role of IMAT are lacking. The aim of this work was to identify novel mechanisms by which IMAT may directly impact skeletal muscle metabolism. METHODS/STUDY POPULATION: We quantified the secretome of IMAT, subcutaneous adipose tissue (SAT), and visceral adipose tissue (VAT) to determine if there are differences between depots in the secretion of cytokines, eicosanoids, FFAs and proteins that influence metabolic function. SAT and VAT biopsies from patients undergoing laparoscopic bariatric surgery and IMAT extracted from vastus lateralis biopsies of individuals with Obesity were cultured for 48 hours in DMEM, and the conditioned media was analyzed using nanoflow HPLC-MS, multiplex ELISAs and LC/MS/MS for proteins, cytokines and eicosanoids/FFA, respectively. RESULTS/ANTICIPATED RESULTS: IMAT secretion of various extracellular matrix proteins (fibrinogen-β, collagenV1a3, fibronectin) was significantly different than VAT and SAT. Pro-inflammatory cytokine secretion of IFNg, TNFa, IL-8 and IL-13 from IMAT was higher than VAT and significantly higher than SAT (p < 0.05). IMAT secretes significantly more pro-inflammatory eicosanoids TXB_2_ and PGE_2_ than VAT (p = 0.02, 0.05) and SAT (p = 0.01, 0.04). IMAT and VAT have significantly greater basal lipolysis assessed by FFA release rates compared to SAT (p = 0.01, 0.04). DISCUSSION/SIGNIFICANCE OF IMPACT: These data begin to characterize the disparate secretory properties of SAT, VAT and IMAT and suggest a metabolically adverse secretome of IMAT, that due to its proximity to skeletal muscle may play an important functional role in the pathogenesis of insulin resistance and type 2 diabetes.

